# Normal Uterus and Ovary in the Indonesian Population: A Comprehensive Analysis

**DOI:** 10.1089/whr.2025.0023

**Published:** 2025-05-12

**Authors:** Andi Darma Putra, Aldi Tamara Rahman, Lasmini Syariatin, Naufal Syafiq Darmawan, Dian Catur Permatasari

**Affiliations:** ^1^ Division of Gynecologic Oncology, Department of Obstetric and Gynecology, Faculty of Medicine, Universitas Indonesia, Cipto Mangunkusumo Hospital, Central Jakarta, Indonesia.; ^2^ Dopamine Science Institute, Depok, Indonesia.; ^3^ Assistant Division of Gynecologic Oncology, Department of Obstetric and Gynecology, Cipto mangunkusumo Hospital, Central Jakarta, Indonesia.

**Keywords:** ultrasonography, uterus, ovary, menopause, Indonesia, retrospective studies

## Abstract

**Objective:**

This research seeks to establish comprehensive baseline data on the normal anatomical features of the uterus and ovaries in a cohort of healthy Indonesian subjects.

**Methods:**

This research was a retrospective study employing 500 ultrasonography results from various gynecological examinations conducted between 2021 and 2024. The ultrasound record serves as a basis for diagnosis and the definition of inclusion criteria. A morphometric analysis will be performed on the uterus and ovaries of the subject.

**Results:**

The study sample comprised 121 healthy women from reproductive age to menopause, examined using ultrasonography. Significant differences in uterine volume (*p* < 0.05) were observed between the 21–30 age group and the 31–40, 41–50, 51–60, and >60 age groups. Furthermore, differences in ovarian volume were observed between the right and left ovaries, as well as between women of reproductive age and those who are menopausal.

**Conclusion:**

This study on ovarian and uterine volumes in Indonesian women offers significant insights into reproductive health, highlighting age-related changes and prospective improvements in diagnostic accuracy.

## Introduction

The examination of uterine and ovarian anatomy is essential for evaluating women’s reproductive health and identifying various gynecological conditions.^1,2^ Establishing normative values for uterine and ovarian dimensions can assist clinicians in recognizing anomalies that may indicate underlying diseases, including premature ovarian insufficiency, functional hypothalamic amenorrhea, polycystic ovary syndrome, and endometriosis.^3–6^ However, there is a lack of comprehensive data regarding the anatomical characteristics of these organs in the Indonesian population.

The uterus and ovaries are essential reproductive organs in females, responsible for gamete production, sex hormone regulation, and providing support of fetal development and delivery.^7,8^ Their functions, such as gamete production, sex hormone regulation, and support of fetal development and delivery, are regulated by reproductive hormones, including estrogen, progesterone, and gonadotropins, which are produced by the pituitary gland and ovaries.^9^ The organs include internal structures such as the ovaries, oviducts, uterus, vagina, external genitalia, and mammary glands.^10^ The ovaries, as the primary reproductive organs, play a crucial role in hormone production and the maturation of oocytes. They are located adjacent to the uterus, facilitating these essential reproductive processes. Despite comprehensive global research, the structural characteristics of these organs within the Indonesian population remain poorly explored. This highlights the importance of localized research to guide clinical practices and improve patient outcomes.

Uterine volume differences among ethnic groups suggest that Indonesian women may display unique morphological characteristics.^11^ For instance, the average uterine volume in European women ranges from 38 to 65 mL, whereas in Asian women, it spans from 24 to 50 mL during adolescence.^12,13^ The dimensions, morphology, and spatial orientation of the ovaries can vary due to genetic, environmental, or lifestyle influences. Variations in the thickness and histological composition of the endometrium could lead to differences that may impact reproductive function and fertility, underscoring the importance of understanding endometrial characteristics.^14^ The distinctions highlight the significance of conducting population-specific studies to improve reproductive health strategies.

Considering the important roles of the uterus and ovaries, precise evaluation of their dimensions and morphology is vital for understanding reproductive health and diagnosing associated diseases. Ultrasonography is crucial in determining the size and structure of the uterus and ovaries. This imaging modality minimizes patient exposure to ionizing radiation, facilitates multisectional organ scanning, and offers a cost-effective and convenient diagnostic alternative.^15^ Vaginal and abdominal sonography accurately assesses uterine dimensions, allowing examination to a depth of 10 cm when the patient has a full bladder. A transmitter featuring a long focal center is essential, with real-time scanning identified as the preferred approach.^16^ Moreover, specific sonographic measurements may predict the start of conditions like uterine myoma and adenomyosis, highlighting the importance of this modality in gynecological evaluations.^17^

This study aims to investigate the normal anatomical characteristics of the uterus and ovaries in a group of healthy individuals from Indonesia, thus addressing a gap in the existing literature. We conducted a comprehensive analysis of the uterus and ovaries in a sample of Indonesian individuals to fill this knowledge gap. This study employed various imaging modalities, including magnetic resonance imaging and ultrasonography, to assess the morphology and microstructure of the organs. The results of our study provide substantial insights into the normal anatomy of the uterus and ovaries in the Indonesian population. The results have considerable implications for the diagnosis and management of reproductive health issues, as well as for the development of targeted medical treatments.

## Material and Methods

### Ethical clearance

Approval was obtained from the Ethics Committee of the Faculty of Medicine, Universitas Indonesia, prior to the commencement of data collection (Ethical Clearance Number: KET-12/UN2.F1/ETIK/PPM.00.02/2025). A waiver of informed consent was granted due to the retrospective nature of the study, which involved data collection from patients’ medical records.

### Study design

This observational study includes female patients from various age groups, categorized into five segments: 20–30, 30–40, 40–50, 51–60, and >60 years. Morphometric analysis was performed on the patient’s uterus and ovaries. A retrospective medical record audit was conducted as part of a descriptive study between 2021 and 2024 at Cipto Mangunkusumo Hospital, Central Jakarta, and Pantai Indah Kapuk Hospital, North Jakarta.

### Ultrasound data collection and validation

Ultrasonography was conducted using the Mindray Resona 7 system with a V11-3HU probe (Mindray, United States) and Voluson Expert 22 with Convex Matrix Array Volume RM7C probe (GE Healthcare, Austria). The probes are equipped with a microconvex ultrasound transducer designed for intracavitary applications, specifically in obstetrics and gynecology. The system has an interactive touchscreen interface, two-dimensional scanning mode, Doppler imaging, elastography, and volumetric imaging. It features an integrated measurement calculator, automated diagnostics, and tailored image processing software to improve diagnostic efficiency and accuracy.

The morphological volumetric analysis of uterus and ovaries was measured using 3—11 MHz frequency range. The longitudinal measurement was for length and thickness measurement, while their width was assessed transversely. Once the measurements for length, thickness, and width were obtained, the ultrasound device automatically calculated the volume of each organ. The obtained value was further compared for validation with the following formula: Volume(V)=Length(L)×Width(W)×Height(H)×0,5226.^18–20^

### Exclusion and inclusion criteria

#### Inclusion criteria

1. Patients do not exhibit abnormal gynecological issues.
2. Patients have no history of prior pathological gynecological issues.
3. Patients have no records of genetic anomalies affecting the morphology and dimensions of the uterus and ovaries.
4. The patient underwent a gynecological ultrasound examination and was deemed normal.

#### Exclusion criteria

1. Patients who got diagnosed with gynecological issues.
2. Patients possessed a history of prior pathological gynecological conditions.
3. Patients possessed genetic anomalies that influence the morphology and dimensions of the uterus and ovaries.

### Statistical analysis

Descriptive statistics were conducted using IBM SPSS Statistics for Windows, Version 29.0 (Armonk, NY: IBM Corp). Statistical analysis was performed using the Kruskal–Wallis test to compare multiple groups. When significant differences were observed (*p* < 0.05), *post hoc* analysis was carried out using the Mann–Whitney U test to determine specific group differences.

## Results

**FIG. 1.**
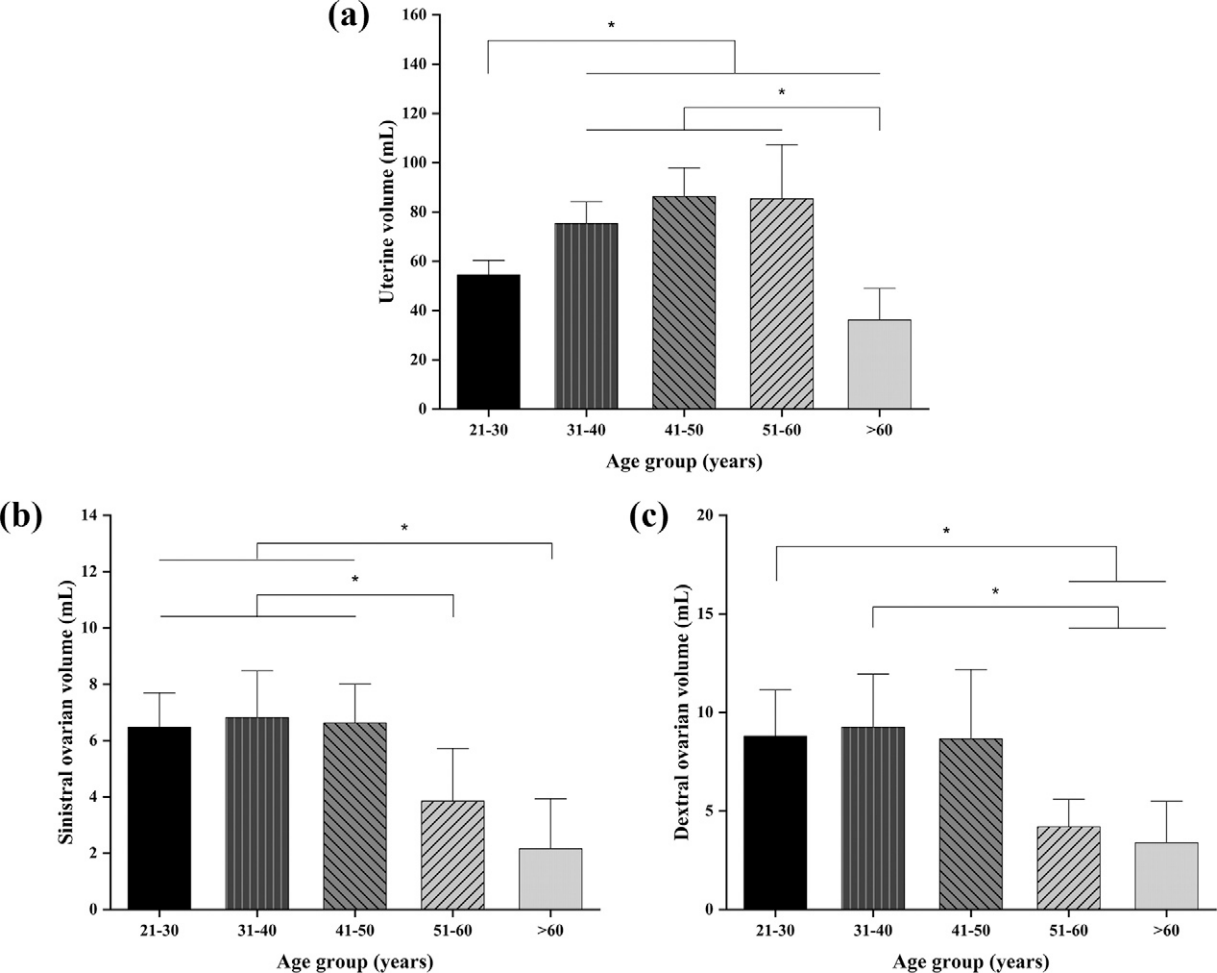
Measurement of **(a)** uterine, **(b)** left (sinistral) ovarian, and **(c)** right (dextral) ovarian. * is significant based on Mann–Whitney U *post hoc* test (*p* < 0.05).

**FIG. 2.**
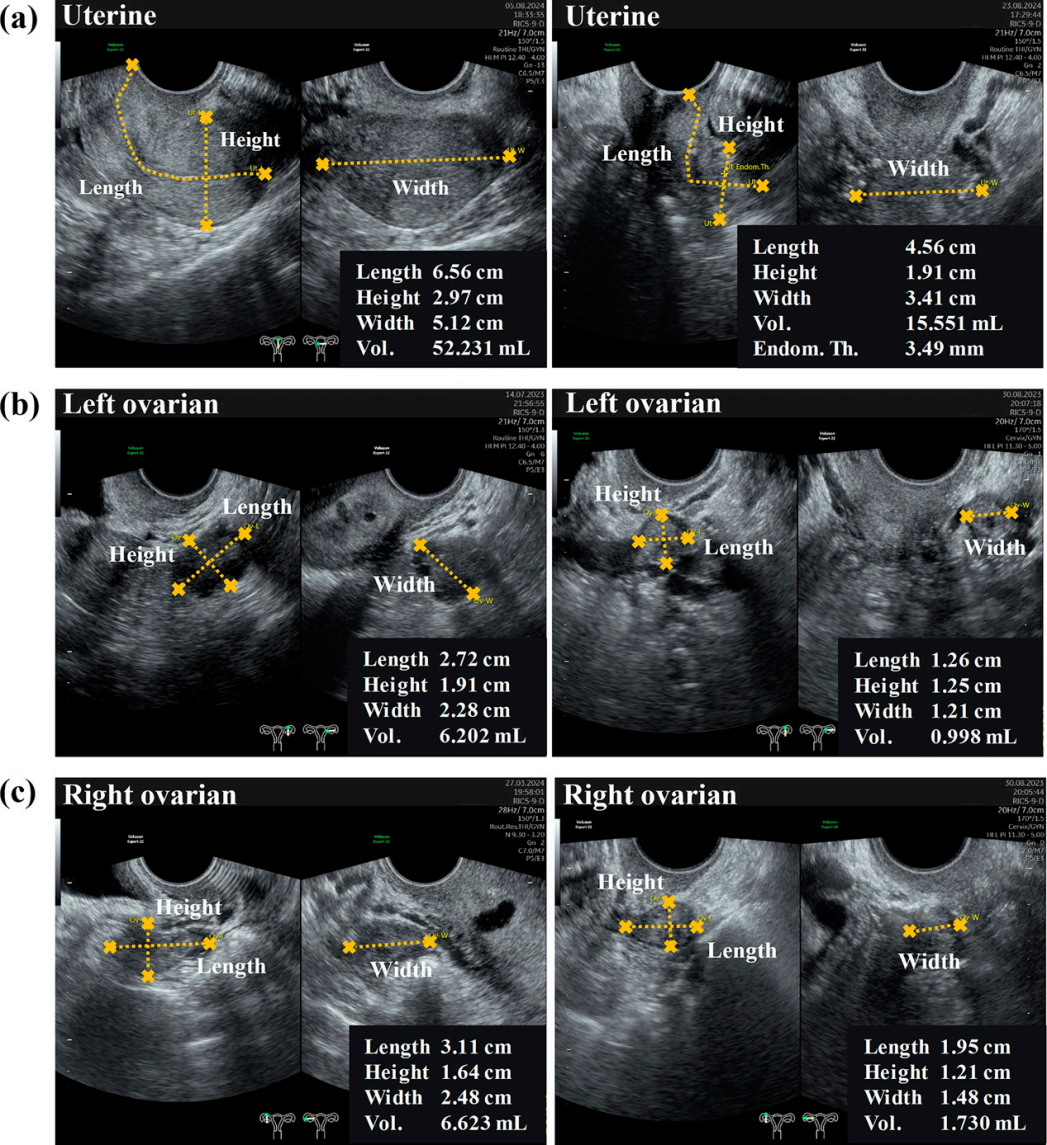
Ultrasonographic images of **(a)** uterus, **(b)** left ovary, and **(c)** right ovary. The left column shows organs in reproductive-age women (31–40 years old), while the right column depicts organs in postmenopausal women (>60 years old).

Out of 500 patients who presented for obstetric and gynecological ultrasound services, only 121 women met the eligibility criteria based on the predetermined inclusion and exclusion criteria. Figures 1a and 2a illustrate the mean uterine volumes across different age groups: 21–30 years (54.53 ± 2.95 mL), 31–40 years (75.53 ± 4.51 mL), 41–50 years (86.31 ± 5.90 mL), 51–60 years (85.32 ± 11.21 mL), and >60 years (36.18 ± 6.52 mL). Our analysis revealed statistically significant differences (*p* < 0.05) in uterine volume between the 21–30 age group and the 31–40, 41–50, 51–60, and >60 age groups. In addition, significant differences were observed between the >60 age group and the 21–30, 31–40, and 51–60 age groups.

As shown in Figures 1b and 2b, the mean volumes for the left ovary in normal women across the same age groups were 6.47 ± 0.62 (21–30 years), 6.82 ± 0.84 (31–40 years), 6.63 ± 0.70 (41–50 years), 3.85 ± 0.95 (51–60 years), and 2.16 ± 0.90 mL (>60 years). Differences in ovarian volume between the right and left ovaries were observed. In the left ovary, significant differences were found between women aged 51–60 and >60 years compared with those in the 21–30, 31–40, and 41–50 age groups.

For the right ovary (Fig. 1c and 2c), the mean volumes of the right ovary in normal women were observed across various age groups, measured in milliliters (mL): 8.80 ± 1.19 (21–30 years), 9.25 ± 1.37 (31–40 years), 8.66 ± 1.79 (41–50 years), 4.20 ± 0.71 (51–60 years), and 3.39 ± 1.06 (>60 years). Significant differences were found between women aged 51–60 and >60 years compared with those in the 21–30 and 31–40 age groups.

In conclusion, the right ovary exhibited significant differences primarily between the 51–60 and >60 age groups when compared with the 21–30 and 31–40 age groups, while variations in the left ovary were observed throughout a wider spectrum of age groups.

## Discussion

Understanding morphometric variations of an organ is essential for clinicians, surgeons, and radiologists as a reference for accurate diagnoses.^21^ Furthermore, understanding the normal range of organ sizes specific to a population is crucial for precise assessment of women within that population.^22^ Previous studies have reported differences in organ dimensions across different populations. For example, the average uterine volume for women aged 21–30 years in the world is ∼73.50 mL,^23^ whereas in Nigeria, it is reported to be 41.14 ± 6.68 mL for the same age group.^24^ These differences can be attributed to several factors, including genetics, environmental influences, and dietary habits across populations.^25,26^ Therefore, our study aims to reveal the morphometric normal ranges of the uterus and ovaries in a population of healthy Indonesian women, addressing this critical need for population-specific data.

We observed significant changes in uterine volume between elderly women (>60 years) and those in young adulthood and middle age (45–59 years). Figure 1c illustrated a trend of increasing uterine volume from ages 21–30 to 41–50 years, followed by a decline in volume at ages 51–60 and >60 years. This pattern corresponds with previous research, indicating that uterine volume begins to decrease before the final menstrual period and subsequently decreases rapidly.^27^ This phenomenon is attributed to uterine atrophy, caused by several factors: the hypoestrogenic condition following menopause, changes in endometrial tissue in which postmenopausal women exhibit low or no fluid in the uterine cavity (resulting in epithelial micro erosions and inflammation), and subsequent vascular fibrosis and reduced blood flow, all contributing to uterine shrinkage. These findings align with the known understanding of postmenopausal changes in uterine morphology.^27,28^

Similarly, we observed a dramatic decrease in ovarian volume in normal women >50 years of age (Fig. 1b and c). This finding is consistent with the study conducted by Pavlik and others in 2000, which reported a significant decline in ovarian volume between the ages of 30 and 70 years, with the average ovarian volume in premenopausal women exceeding that of postmenopausal women. This is a common phenomenon observed in all women, where ovarian size is correlated with the number of follicles present in the ovary.^29^ Furthermore, with increasing age, the number of follicles in the ovaries decreases progressively, leading to menopause, which is characterized by the total depletion of follicles, reduced secretion of estrogen and inhibin B, and higher secretion of follicle stimulating hormone as a negative feedback response. Wen and others reported in 2021 that the loss of follicles directly contributes to the reduction in ovarian size.^30^ As previously mentioned, the volume of the right ovary was consistently larger than that of the left ovary among those in reproductive age. This finding may agree with several explanations. The right ovary has a locational advantage due to the left side of the pelvis having more restricted space than the right side. Secondly, the venous drainage system differs between the two ovaries: the right ovarian vein drains directly into the inferior vena cava, while the left ovarian vein drains into the renal vein. This difference is thought to affect the efficiency of the circulatory system and nutrient transport. Consequently, these anatomical and vascular variations are believed to be responsible for the size difference between the right and left ovaries.^31,32^

## Conclusions

This comprehensive study has successfully established normative values for uterine and ovarian volumes in the Indonesian female population, addressing a significant gap in regional reproductive health data. The findings serve as a valuable reference point in the diagnostic procedure for various gynecological conditions. Notably, this research has demonstrated statistically significant differences in uterine and ovarian dimensions between women of reproductive age and postmenopausal women. These age-related variations in reproductive organ size offer important insights into the physiological changes that occur during a woman’s lifespan. This population-specific data enhances the precision and cultural relevance of reproductive health evaluation, potentially improving diagnostic accuracy and patient care in Indonesia.

## Data Availability

The datasets used and/or analyzed during the current study are available from the corresponding authors on reasonable request.

## Abbreviations Used

H: Height
L: Length
MHz: Megahertz
mL: Milliliter
NY: New York
SPSS: Statistical Package for the Social Sciences
V: Volume
W: Width
